# Control of cardiovascular risk factors and its determinants in the general population– findings from the STAAB cohort study

**DOI:** 10.1186/s12872-017-0708-x

**Published:** 2017-11-02

**Authors:** Theresa Tiffe, Martin Wagner, Viktoria Rücker, Caroline Morbach, Götz Gelbrich, Stefan Störk, Peter U. Heuschmann

**Affiliations:** 1 Institute of Clinical Epidemiology and Biometry, University of Würzburg, Würzburg, Germany; 2 Comprehensive Heart Failure Center Würzburg, University of Würzburg, Würzburg, Germany; 3 Department of Medicine I, University Hospital Würzburg, Würzburg, Germany; 4 Clinical Trial Center Würzburg, University Hospital Würzburg, Würzburg, Germany

**Keywords:** Population-based study, Prevalence, Risk factor control, Guideline adherence, Primary prevention

## Abstract

**Background:**

While data from primary care suggest an insufficient control of vascular risk factors, little is known about vascular risk factor control in the general population. We therefore aimed to investigate the adoption of adequate risk factor control and its determinants in the general population free of cardiovascular disease (CVD).

**Methods:**

Data from the Characteristics and Course of Heart Failure Stages A-B and Determinants of Progression (STAAB) Cohort Study, a population-based study of inhabitants aged 30 to 79 years from the general population of Würzburg (Germany), were used. Proportions of participants without established CVD meeting targets for risk factor control recommended by 2016 ESC guideline were identified. Determinants of the accumulation of insufficiently controlled vascular risk factors (three or more) were assessed.

**Results:**

Between December 2013 and April 2015, 1379 participants without CVD were included; mean age was 53.1 ± 11.9 years and 52.9% were female; 30.8% were physically inactive, 55.2% overweight, 19.3% current smokers. Hypertension, dyslipidemia, and diabetes mellitus were prevalent in 31.8%, 57.6%, and 3.9%, respectively. Treatment goals were not reached despite medication in 52.7% of hypertensive, in 37.3% of hyperlipidemic and in 44.0% of diabetic subjects. Insufficiently controlled risk was associated with male sex (OR 1.94, 95%CI 1.44–2.61), higher age (OR for 30–39 years vs. 70–79 years 4.01, 95%CI 1.94–8.31) and lower level of education (OR for primary vs. tertiary 2.15, 95%CI 1.48–3.11).

**Conclusions:**

In the general population, prevalence of vascular risk factors was high. We found insufficient identification and control of vascular risk factors and a considerable potential to improve adherence to cardiovascular guidelines for primary prevention. Further studies are needed to identify and overcome patient- and physician-related barriers impeding successful control of vascular risk factors in the general population.

**Electronic supplementary material:**

The online version of this article (10.1186/s12872-017-0708-x) contains supplementary material, which is available to authorized users.

## Background

Cardiovascular disease (CVD) represents the major cause of hospital admission, disability in middle-aged and older patients, and remains the leading cause of death (40%) in Germany [1, 2]. The burden of CVD is maintained by the high prevalence of modifiable vascular risk factors in the population including an unhealthy lifestyle [3]. Several national and international guidelines describe the principles of cardiovascular prevention in people without established CVD (primary prevention) [4–7]. Most guidelines tailor their recommendations according to the estimated absolute CVD risk (e.g. by applying risk scores) including the adoption of a healthy lifestyle in low risk persons (e.g. prudent eating habits, non-smoking, avoiding obesity, regular physical activity), and uptake of drug medication in the asymptomatic high risk population [4].

Previous studies reporting on the quality of adequate cardiovascular (CV) risk factor control in primary prevention were frequently based on samples derived from primary care. These studies suggested an insufficient implementation of CVD prevention strategies including management of obesity, blood pressure (BP), and lipid and glucose metabolism [8–15]. Little, however, is known about of the control of risk factors CVD in population-based samples [16]. This is important as estimates originating from studies in primary care may not be representative of the general population because individuals attending a primary care physician usually have a reason to do so. Such selection and indication bias will profoundly affect prevalence estimates.

Therefore, we assessed in subjects without established CVD sampled from the general population of Germany the prevalence of CV risk factors, the frequency of inadequate risk factor control, and factors determining the accumulation of insufficiently controlled risk.

## Methods

### Study population and recruitment

The methodology of Characteristics and Course of Heart Failure Stages A-B and Determinants of Progression (STAAB) Cohort Study has been published previously [17]. Briefly, STAAB was initiated in Dec 2013 to determine the prevalence and natural course of the early stages of heart failure in a representative sample (*n* = 5000) of the general population of Würzburg aged 30–79 years. Participants undergo a detailed examination at the joint survey unit of the Comprehensive Heart Failure Center (CHFC) and the Institute of Clinical Epidemiology and Biometry (ICE-B) of the University of Würzburg, Germany. The current report is based on the first planned interim-analysis of STAAB including *n* = 1468 participants, who had been enrolled by April 2015 (positive response rate 32.6%) [17]. We excluded *n* = 89 (6.1%) participants with established CVD defined by self-reported history of coronary artery disease (*n* = 56), peripheral artery disease (*n* = 11), stroke (*n* = 28). Hence, the current report is based on *n* = 1379 subjects.

### Assessment of cardiovascular risk factors

Sociodemographic status, information on smoking and on physical activity was obtained via face-to-face interview. History of CVD and medication intake was assessed by study physicians. BP values were given as median of up to three valid measurements. Low density liproprotein (LDL) cholesterol and glycated hemoglobin levels (HbA1c) levels were measured at the day of examination at the Central Core Laboratory of the University Hospital of Würzburg.

### Treatment goals for cardiovascular risk factors

We identified the proportions of participants achieving the defined goals for risk factor control according to the most recent European Guidelines on CVD prevention in clinical practice (version 2016) [4] including: target BP <140/90 mmHg (<140/85 mmHg in subjects with DM type 2; <130/80 mmHg in DM type 1); target LDL cholesterol <115 mg/dl (<100 mg/dl in diabetics and participants at high risk [for definition, see below]); absence of tobacco abuse (self-reported in a structured interview); absence of overweight (body mass index ≤25 kg/m^2^); absence of diabetes mellitus (DM; no self-reported DM and HbA1c <6.5%); adequate glycemic control in treated diabetics (HbA1c <7.0%); and physical activity (≥150 min/week moderate activity or ≥75 min/week strenuous activity, operationalized by the IPAQ questionnaire [18]). All risk factors above recommended target that were not self-reported (high BP, high LDL-cholesterol and diabetes) were defined as unreported. For the definitions of the six uncontrolled CV risk factors and their subgroups, see: Additional file 1.

### Determinants of accumulation of uncontrolled risk factors

We identified a priori a set of covariates that were potentially related to the control of CV risk factors including: age, sex, markers of socioeconomic status (level of education and income), and marital status [19–22]. In addition, we performed a sensitivity analysis investigating if the predictors of three or more uncontrolled risk factors differed by sex.

### Absolute cardiovascular risk estimation with systematic COronary risk evaluation (SCORE)

To assess differences in prevalence of risk factor control across different CV risk groups, we applied the recently updated SCORE algorithm for Germany predicting 10-year risk of CV death [23]; SCORE has been derived from subjects without a constellation indicating increased vascular risk (as prior CVD or DM). The SCORE equation weights the following factors: age (40–69), sex, current smoking, systolic BP, and total cholesterol. For the present analyses, our sample was categorized according to thresholds recommended by the European Society of Cardiology into “low risk” (SCORE <1%), “moderate risk” (SCORE ≥1% and <5%), and “high to very risk” (SCORE ≥5%) [4, 24].

### Data analysis

We calculated mean standard deviation (±SD) and median (quartiles) for continuous variables and proportions (%) for categorical variables. In univariate analyses, Fisher’s exact test or χ^2^- test for categorical and binary variables, Mann-Whitney U-test for continuous variables (non-normal distributions) and Kruskal-Wallis test were used as appropriate. Accumulation of uncontrolled risk factors (RR ≥140/90 mmHg [≥130/80 mmHg for subjects with diabetes]; LDL cholesterol ≥115 mg/dl; tobacco abuse (self-reported); overweight [body mass index >25 kg/m^2^]; DM [HbA1c >6.5%] and physical inactive [<150 min/week moderate or <75 min/week strenuous activity]) was categorized by the median sum of uncontrolled risk factors in our population (0–2 vs. 3–6). We calculated odds ratios (OR) for the association of age, sex, education and marital status with accumulation of risk factors by multivariable logistic regression. *P*-values <0.05 were considered statistically significant. Analyses were performed with IBM SPSS Statistics 23 (IBM® SPSS® Statistics Version 23).

## Results

### Characteristics of the study participants and prevalence of CV risk factors

Characteristics of the study population and prevalence of CV risk factors by sex are displayed in Table 1. Mean age was 53.1 years (SD 11.9), 52.9% were females. As compared to women, men reported a longer duration of education and a higher household income per month.  More men than women were married (*p* <0.001), whereas more women than men were divorced ore widowed (both *p* <0.001).

**Table 1:** Sociodemographic status and control of risk factors stratified by sex

	N_Total_	Female	Male	*P*-value
	1379	729 (52.9)	650 (47.1)	
*Age group in years*				0.62
30–39	166 (12.0)	82 (11.2)	84 (12.9)	
40–49	417 (30.2)	226 (31.0)	191 (29.4)	
50–59	339 (24.6)	185 (25.4)	154 (23.7)	
60–69	340 (24.7)	180 (24.7)	160 (24.6)	
70–79	117 (8.5)	56 (7.7)	61 (9.4)	
*Highest education in years*				<0.001
Primary (<10 yrs)	331 (24.1)	163 (22.4)	168 (26.0)	
Secondary (10 yrs)	411 (29.9)	257 (35.3)	154 (23.8)	
Tertiary (12 yrs)	621 (45.2)	301 (41.3)	320 (49.5)	
Unclassified	12 (0.9)	7 (1.0)	5 (0.8)	
*Marital status*				<0.001
Single	337 (24.5)	177 (24.3)	160 (24.7)	
Married	811 (59.0)	396 (54.4)	415 (64.1)	
Divorced	157 (11.4)	104 (14.3)	53 (8.2)	
Widowed	70 (5.1)	51 (7.0)	19 (2.9)	
*Household net income per month in Euro*				<0.001
<1500	168 (13.2)	112 (16.9)	56 (9.1)	
1500 to <2900	458 (35.9)	259 (20.3)	199 (32.4)	
2900 to <5000	421 (33.0)	192 (29.0)	229 (37.3)	
>5000	229 (17.9)	99 (15.0)	130 (21.2)	
*BP* ^*a*^ *(mmHg)*				
Systolic	129.0 (118.0; 142.0)	123.0 (113.0; 138.0)	133.5 (123.0; 144.0)	<0.001
Diastolic	79.0 (72.5; 85.5)	76.5 (70.5; 83.5)	81.0 (75.5; 88.0)	<0.001
*High BP levels*				
Self-reported hypertension	546 (39.6)	282 (38.7)	264 (40.6)	0.46
BP ≥140/90 mmHg	433 (31.8)	177 (24.7)	256 (39.6)	<0.001
Unreported high BP above target	156 (36.0)	47 (26.6)	109 (42.6)	0.001
Antihypertensive medication	372 (27.0)	197 (27.0)	175 (27.0)	0.90
High BP level despite medication^e^	193 (52.7)	94 (48.7)	99 (57.2)	0.12
Diabetics treated with antihypertensive agents	38 (67.9)	17 (68.0)	21 (67.7)	0.98
High BP level despite medication^e^ in diabetics				
Type 1	2 (5.6)	1 (6.7)	1 (4.8)	0.76
Type 2	16 (44.4)	6 (40.0)	10 (47.6)	0.62
Diabetes mellitus				
Self-reported diabetes	79 (5.7)	43 (5.9)	36 (5.5)	0.77
HbA1c >6.5%	46 (3.5)	22 (3.2)	24 (3.9)	0.50
Unreported DM above target	13 (28.3)	7 (31.8)	6 (25.0)	0.75
Antidiabetic medication	56 (4.1)	25 (3.4)	31 (4.8)	0.20
HbA1c ≥ 7% despite antidiabetics^e^	22 (44.0)	9 (39.1)	13 (48.1)	0.52
*LDL-cholesterol* ^b^ *(mg/dl)*	120.0 (100.0; 146.0)	117.5 (96.8; 146.0)	123.0 (105.5; 146.0)	<0.01
*High LDL-cholesterol levels*				
Self-reported hyperlipidemia	488 (35.4)	247 (33.9)	241 (37.1)	0.22
LDL-C >115 mg/dl	753 (57.6)	365 (52.9)	388 (62.8)	<0.001
Unreported high LDL-C levels above target	408 (54.2)	187 (51.2)	221 (57.0)	0.12
Lipid-lowering agents	112 (8.1)	47 (6.4)	65 (10.0)	0.02
High LDL-C level despite medication^e^	38 (37.3)	18 (42.9)	20 (33.3)	0.33
Lipid lowering treated diabetics	22 (39.3)	8 (32.0)	14 (45.2)	0.32
High LDL-C level despite medication^e^ in diabetics	4 (21.1)	2 (28.6)	2 (16.7)	0.84
*Physically inactive*	323 (30.8)	180 (33.1)	143 (28.2)	0.08
*Overweight*				<0.001
BMI^c^ (kg/m^2^)	25.5 (23.0; 28.9)	24.7 (21.9; 28.5)	26.3 (24.2; 29.1)	<0.001
BMI > 25 kg/m^2^	751 (55.2)	336 (46.7)	415 (64.7)	<0.001
BMI > 25 kg/m^2^ despite physical activity	393 (54.9)	162 (45.4)	231 (64.3)	<0.001
*Current smoking*	266 (19.3)	121 (16.6)	145 (22.3)	<0.01
*Insufficiently controlled CV risk factors* ^*d*^				<0.001
0–2	639 (65.8)	361 (72.3)	278 (58.9)	
3–6	322 (23.2)	138 (27.7)	194 (41.1)	

Data are count (percent) or median (quartiles). Analyses are restricted to patients without missing values in respective variables.

^a^Blood pressure

^b^Low density lipoprotein- Cholesterol

^c^Body mass index

^d^Cardiovascular risk factors

^e^Proportion addressed at participant being treated

Of all participants, 19.3% reported current smoking (Table 1), with a significant preponderance in men. Overweight and high BP were significantly more present in men compared to women, as were LDL cholesterol levels. Prevalence of self-reported hypertension, hyperlipidemia and diabetes was independent from sex.

### Achievement of recommended treatment goals and its determinants

CV pharmacotherapy was reported in 422 individuals (30.7%), with no difference between women and men, but a significant greater proportion observed at higher age (*p* <0.001).

### Hypertension

In the total sample 433 participants (31.8%) had BP levels above the recommended target of 140/90 mmHg (Table 1). More men were detected with higher BP levels as compared to women (39.6% vs. 24.7%, respectively; *p* <0.001). Amongst subjects with raised BP levels, 156 (36.0%) had not been diagnosed by previous visits to a physician (women: 26.6% vs. men: 42.6%, *p* = 0.001). Amongst 372 (27.0%) subjects receiving antihypertensive medication, 193 (52.7%) did not achieve recommended BP targets (men 48.7% vs. women 57.2%, *p* = 0.12).

### Diabetes mellitus

At the study visit, 46 subjects (3.5%) had an HbA1c value >6.5%, with no sex-specific differences (*p* = 0.50), while 13 (28.3%) were diagnostically naïve. Of the 56 (4.1%) participants receiving antidiabetic medication, 22 (44.0%) did not reach the recommended target (HbA1c <7.0%). Of 38 (67.9%) diabetic participants treated for hypertension, 16 (44.4%) subjects with DM type 2 were above the recommended BP of 140/85 mmHg, and 2 subjects (5.6%) >130/80 mmHg for DM type 1. Of subjects treated for hyperlipidemia, 4 (21.1%) had LDL cholesterol levels >100 mg/dl, with no differences beteen sexes.

### Hyperlipidemia

More than a half of the participants (57.6%) had LDL cholesterol levels equal or above the target of 115 mg/dl (men 62.8% vs. women 52.9%, *p* <0.001). Amongst those, 408 subjects (54.2%) were unaware of this constellation; 112 participants took lipid-lowering medication, with 38 individuals (37.3%) above the recommended target despite medication.

We found the highest proportion of unreported risk factors at the age of 30–39 years for high BP levels (76.5%) and high LDL cholesterol (78.0%), and at the age of 60–69 years for HbA1c >6.5% (43.5%).

### Determinants of accumulation of uncontrolled risk factors

In the total sample, the median number of uncontrolled risk factors was 2 (range 0–6; quartiles 1, 3). Only a small proportion, i.e. 11.4%, was lacked any risk factor. In the multivariable model, probability for having three or more uncontrolled risk factors was associated with male sex (OR 1.94, 95%CI 1.44–2.61), lower level of education (OR for primary vs. tertiary: 2.15, 95%CI 1.48–3.11) and higher age (OR for 30–39 years vs. 70–79 years: 4.01, 95%CI 1.94–8.31; Table 2). In a sensitivity analysis, no major differences became apparent between men and women regarding the predictors of 3 or more uncontrolled risk factors [for details refer to Additional file 2].

**Table 2:** Relative risk (odds ratio) for exhibiting 3–6 insufficiently controlled cardiovascular risk factors (referent: 0–2 risk factors)

Variables	Odds ratio (95%CI)	*P*-value
*Sex*		<0.001
Female	1	
Male	1.94 (1.44; 2.61)	
*Age group in years*		<0.001
30–39	1	
40–49	1.77 (1.01; 3.09)	
50–59	2.10 (1.18; 3.72)	
60–69	3.26 (1.84; 5.78)	
70–79	4.01 (1.94; 8.31)	
*Highest education in years*		<0.001
Tertiary	1	
Secondary	1.35 (0.95; 1.92)	
Primary	2.15 (1.48; 3.11)	
Household net income per month in Euro*		0.27
≥2300	1	
<2300	1.21 (0.86; 1.769)	
Marital Status*		0.48
Married	1	
Single	0.82 (0.57; 1.23)	
Divorced	1.27 (0.78; 2.01)	
Widowed	1.07 (0.54; 2.14)	

Insufficiently controlled cardiovascular risk factor: value above target, independent of patient awareness and current pharmacotherapy, adjusted for sociodemographic status.

*Odds ratio given before being removed from model

### 10-year risk estimation for fatal CVD by SCORE

**Fig. 1:**
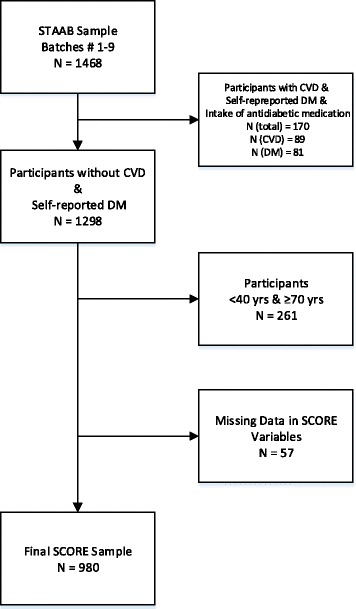
Sample Selection for SCORE estimation

For this analysis subjects with DM, CVD, and those younger than 40 years or older than 69 years were excluded (Fig. 1). Hence, SCORE values could be calculated in 980 subjects. Median SCORE was 1.0 (quartiles 1, 2), and subjects were categorized into low, medium, high to very high risk categories in 56.6%, 35.8%, and 7.5%, respectively (Table 3).

**Table 3:** Sociodemographic status and control of cardiovascular risk factors stratified by SCORE

	N_Total_	Total cardiovascular risk (SCORE)			
	980	<1% (low) 555 (56.6)	≥1 to <5% (medium) 351 (35.8)	≥5% (high to very high) 74 (7.5)	*p*-value
*Gender*					<0.001
Female	523 (53.4)	374 (67.4)	146 (41.6)	3 (4.1)	
Male	457 (46.6)	181 (32.6)	205 (58.4)	71 (95.9)	
*Age in years*					<0.001
40–49	377 (38.5)	359 (64.7)	18 (5.1)	0 (0.0)	
50–59	308 (31.4)	171 (30.8)	133 (37.9)	4 (5.4)	
60–69	295 (30.1)	25 (4.5)	200 (57.0)	70 (94.6)	
*Highest education* *in* *years*					0.001
Primary (< 10 yrs)	223 (22.8)	96 (17.3)	104 (29.7)	23 (31.5)	
Secondary (10 yrs)	319 (32.7)	188 (33.9)	111 (31.7)	20 (27.4)	
Tertiary (12 yrs)	426 (43.6)	265 (47.8)	132 (37.7)	29 (39.7)	
unclassified	9 (0.9)	5 (0.9)	3 (0.9)	1 (1.4)	
*Marital status*					0.13
Single	208 (21.2)	132 (23.8)	67 (19.1)	9 (12.2)	
Married	610 (62.3)	337 (60.8)	219 (62.4)	54 (73.0)	
Divorced	122 (12.5)	67 (12.1)	47 (13.4)	8 (10.8)	
Widowed	39 (4.0)	18 (3.2)	18 (5.1)	3 (4.1)	
*Net income per month in Euro*					0.02
<1500	109 (12.0)	48 (9.3)	47 (14.5)	14 (20.0)	
1500 to <2900	297 (32.6)	163 (31.6)	111 (37.4)	123 (32.9)	
2900 to <5000	311 (34.1)	182 (35.3)	111 (34.2)	18 (25.7)	
>5000	194 (21.3)	123 (23.8)	56 (17.2)	15 (21.4)	
*BP* ^*a*^ *(mmHg)*					
Systolic	128.0 (118.0; 141.0)	122.5 (114.5; 132.5)	135.5 (124.0; 148.0)	149.3 (137.8;161.9)	<0.001
Diastolic	79.5 (73.0; 86.4)	77.5 (71.5; 84.5)	81.5 (75.0; 88.5)	85.0 (80.0; 90.9)	<0.001
*High BP levels*					
Self-reported hypertension	366 (37.3)	154 (27.9)	173 (49.9)	39 (52.7)	<0.001
BP > 140/90 mmHg	302 (30.8)	90 (16.2)	157 (44.7)	55 (74.3)	<0.001
Antihypertensive treatment	238 (24.3)	85 (15.3)	129 (36.8)	24 (32.4)	<0.001
High BP level despite medication^d^	120 (50.4)	28 (32.9)	70 (54.3)	22 (91.7)	<0.001
*LDL- Cholesterol* ^b^ *(mg/dl)*	124 (104.0; 149.0)	118.0 (98.0; 140.0)	131.0 (111.0; 160.0)	127.5 (105.8148.3)	<0.001
*High LDL-C levels*					
Self-reported hyperlipidemia	347 (35.4)	145 (26.8)	173 (50.4)	29 (40.3)	<0.001
LDL ≥ 115 mg/dl	604 (61.6)	304 (54.8)	251 (71.5)	61 (82.4)	<0.001
Lipid-lowering agents	56 (5.7)	15 (2.7)	31 (8.8)	10 (13.5)	<0.001
High LDL-C level despite medication^d^	27 (48.2)	9 (60.0)	14 (45.2)	4 (40.0)	0.54
*Physically * *inactive*	224 (29.5)	137 (31.1)	75 (28.5)	12 (21.4)	0.30
*Overweight*					
BMI^c^ (kg/m^2^)	25.5 (22.8; 28.8)	24.7 (22.1; 28.1)	26.2 (23.7; 29.4)	27.5 (24.7; 29.5)	<0.001
BMI > 25 kg/m^2^	536 (55.1)	260 (47.1)	222 (64.0)	54 (73.0)	<0.001
BMI > 25 kg/m^2^ despite physical activity^d^	294 (55.6)	145 (48.3)	116 (62.7)	33 (75.0)	<0.001
*Current smoking*	201 (20.5)	102 (18.4)	72 (20.5)	27 (36.5)	0.001

Data are count (percent) and median (quartiles). Analyses restricted to patients without missing values in respective variables

^a^Blood pressure

^b^Low density lipoprotein- Cholesterol

^c^Body mass index

^d^Proportion addressed at participant being treated

Participants at high risk were more likely to be male (*p* <0.001) and of older age (p <0.001). Reflecting the risk factors included in the SCORE calculation, those with higher risk more frequently exhibited hypertension and high LDL cholesterol levels (all i <0.001). Lifestyles habits (overweight and smoking), educational level (*p* <0.001) and net income per month (p = 0.02) were also associated with SCORE categories. In participants receiving antihypertensive medication, the proportion not achieving the recommended BP target rose from 16.2% in the low risk group to 74.3% in the high risk group. In participants with elevated LDL cholesterol levels, risk factors were more frequently insufficiently controlled despite medication (low risk 60.0% vs. high risk 40.0%).

## Discussion

In this cross-sectional study from the general population without established CVD, prevalence of modifiable CV risk factors was high, and risk factors were frequently inadequately controlled (median = 2). A substantial number of individuals did not report any CV risk factor, and in a sizeable proportion of participants on guideline-recommended medication, treatment targets were not achieved. For example, over half of participants on antihypertensive medication had elevated BP levels at the interview. In about one third of participants on lipid lowering drugs, LDL cholesterol levels remained inappropriately high, and in more than 40% of patients on antidiabetic treatment DM was inadequately controlled. We also found a high proportion of adverse lifestyle factors such as insufficient physical activity or overweight and obesity. Individuals with a larger number of insufficiently controlled risk factors were more likely to be older and of male sex, and had spent less time in education.

We investigated the accumulation of cardiovascular risk factors and its determinants in a population-based sample in Germany. Previous European studies on this topic mainly recruited within the general practitioner setting or focused on individual risk factors and their control in population samples. Due to differences in study design and study population, various definitions of adequate risk factor control in national and international recommendation for cardiovascular prevention of CVD over time, and variations in study protocols, direct comparisons of our results with previous studies are limited. However, the prevalence figures of investigated cardiovascular risk factors are comparable with previous data from the population based studies in Germany [25–29].

### Control of risk factors in German population-based samples

#### Hypertension

The longitudinal *DEGS1* (German Health Interview and Examination Survey for Adults) and *GNHIES98* (National Health Interview and Examination Surveys 1998) combined data of 2231 individuals, who were normotensive at baseline but developed hypertension in the course of 12 years of follow-up. This analysis also reported lower proportions of individuals receiving antihypertensive medication but not reaching treatment targets in women (41.0%) as compared to men (49.7%). However, in general, BP control in both genders was better than in our sample, and the occurrence of hypertension was related to higher age, BMI, and alcohol consumption [30]. Another longitudinal German study, *KORA* (Cooperative Health Research in the Augsburg Region in South Germany), showed a similar prevalence of hypertension (49.2%) at baseline, but a much lower achievement of treatment targets in patients on antihypertensive pharmacotherapy (29.6% in men; 43.9% in women) [31]. The highest prevalence of hypertension was found in *CARLA* (the Cardiovascular Disease, Living and Ageing in Halle Study in East Germany) with 66.7% in men and 76.9% in women, which might be due to an overall older study population (mean age in women: 50.0 years; mean age in men: 61.3 years) [32].

#### Diabetes mellitus

Within the *KORA study,* a similar self-reported low prevalence in diabetes of 5.6% compared to STAAB with 5.7% was found [33]. *KORA* participants with DM type 2 diabetes showed comparable glycemic control with a trend for secular improvement from 60% in the year 2000 to 71% in the year 2014 [34], respectively (overall medication control in STAAB: 66%). With respect to the increased CV risk in diabetics [4], we assessed the control of other CV risk factors in this group: despite respective medication, we found elevated BP in 52.6% of diabetic STAAB participants and high LDL cholesterol levels in 21.1%, respectively. The *DIAB-CORE study* (The Diabetes Collaborative Research of Epidemiologic Studies), which assessed the prevalence and management of DM related to BP and lipid management, found higher values of uncontrolled or insufficiently treated hypertension (63.6%), and suboptimally controlled dyslipidemia in diabetic patients (42.5%) [31]. A longitudinal study of the *DIAB-CORE* Consortium in 2015 shows still a large proportion of insufficient hypertension control in diabetics (55.0%) [35].

#### Dyslipidemia

The prevalence of dyslipidemia (total cholesterol ≥190 mg/dl or medical diagnosis of dyslipidemia) in *DEGS1* was 65.1% (women 65.7%; men 64.5%), but more than half of the affected in both genders were unreported. In individuals with known dyslipidemia, only 30.8% were on lipid-lowering medication [36]. In our study, prevalence of dyslipidemia (LDL cholesterol >115 mg/dl) was slightly lower (57.6%), and fewer subjects received insufficient pharmacotherapy (37.3%). This might be due to differing definitions of dyslipidemia, or the result of increased efforts regarding CV risk factor control in Germany.

#### Lifestyle factors

Overweight was prevalent in more than half of the study population, which is in line with *DEGS1* Data reporting a BMI ≥25 kg/m^2^ in 53.0% of women and 67.1% of men [37]. Higher values were found in physically inactive participants (defined as <2.5 h/week of moderate intensity), which was reported more frequently in women (84.5%) than in men (74.6%) [38]. We further found a substantially lower prevalence of current smokers compared to other surveys performed in Germany in 2010 [38, 39]. Concordantly, the *SHIP* study (Study of Health in Pomerania) [27] investigating long-term trends in lifestyle-related risk factors reported decreasing prevalence rates for tobacco smoking, overweight, and physical inactivity [40] over time. However, despite promising signals, control of modifiable vascular risk factors in German remains high calling for concerted actions of better guideline implementation in primary prevention.

### Determinants of cadiovasular risk factor control

In our study, accumulation of insufficiently controlled CV risk factors (i.e., three or more) was associated with male sex, higher age and lower level of education. Two settings became apparent: On the one hand side, there was a fairly large proportion of individuals unaware of or not reporting their risk factors in a structured, physician-led interview. Thus, one strategy for achieving better implementation of guidelines could be a more comprehensive screening for CV risk factors in the general population. On the other hand side, we observed a high prevalence of individuals on pharmacotherapy for a CV risk factor ot achieving recommended treatment goals. As a consequence, education of physicians treating towards targets remains tiring but ever so important effort.

There might be a difference in BP assessed by mercury manometers in primary care practices compared to oscillometric techniques used in our study, which could have resulted in lower mean values for systolic and diastolic BP [41]. This would be in line with findings from the *HYDRA* study (Hypertension and Diabetes Risk Screening and Awareness Study), which reported on an insufficient BP control that seemed to be unnoticed by the treating physicians [9]. Another issue might be the mismatch of insufficient knowledge and compliance from patients´ side and the lack of time for a comprehensive evaluation of all guidelines applying for an individual patient combined with explanations and close guidance from physicians´ side [42, 43].

The *LifeLines cohort study* [44] from the Netherlands focused on the use of lipid-lowering drugs in 70,292 participants (mean age 45 years; *n* = 68,954 without CVD and stroke) based on adherence to the national guidelines for CVD prevention [45]. This study found inadequate control in 77% of the 3268 individuals eligible for lipid-lowering medication. In the entire cohort, prevalence of hypertension (19.0%) and DM (1.7%) was comparatively low, while 22% were current smokers [44]. Another study from the Netherlands (*n* = 1,203,290) revealed a rising number of individuals on CV pharmacotherapy with increasing age [46]. In our sample, we observed 422 (30.7%) participants receiving CV pharmacotherapy increasing from 3% in the age group 30–39 years up to 73.5% in the oldest age group 70–79 years. Interestingly, highest frequencies for of unreported high BP (76.5%) and high LDL cholesterol (78.0%) were found at the age group 30–39 years, and for HbA1c >6.5% (43.5%) at the age group 60–69 years. Of those at high CVD risk, 36 (48.6%) had unreported LDL cholesterol levels ≥100 mg/dl. The importance of this finding is illustrated by a recent study determining the characteristics of culprit plaque in young patients who experienced an acute coronary syndrome. There, patients aged <35 years showed a significant higher risk of plaque rupture [47].

### Risk factor control in primary care settings across Europe

Large patient cohort studies from primary care settings like *DETECT* (Diabetes Cardiovascular Risk-Evaluation: Targets and Essential Data for Commitment of Treatment [*n* = 55,518; mean age 53.9 years, 59.3% women]), *HYDRA* (*n* = 45,125; mean age 52.4 years) or *CoRiMa* (The German Coronary Risk Management [*n* = 248,096, mean age 56.5 years, 53.0% women]) in Germany reported prevalences ranging from 33% to 55% for hypertension, 30% to 62% for hyperlipidemia, and 14 to 29% for diabetes [9–11, 14]. Due to different thresholds, the reported prevalence of CV risk factors in previous studies varied substantially: e.g., thresholds for HbA1c in *DETECT* were >6.1% and in CoRiMa ≥6.5%; hyperlipidemia in CoRiMa (prevalence 62.0%) was based on LDL-C depends on individual cardiovascular risk and the number of risk factors, whereas *DETECT* (prevalence 29.1%) used a threshold of LDL cholesterol >115 mg/dl. As expected, achievement of treatment goals heavily depends on these definitions and are therefore difficult to compare. Treatment goals were achieved in *CoRiMa* for LDL cholesterol in 29%, for BP in 28%, for HbA1c ≥6.5% and/or use of antidiabetic medication in 36%. *HYDRA*, by contrast reported a considerably lower proportion of adequate medication control in only 19% of hypertensive patients taking into account that blood pressure was not standardized an measured in routine care, which may lead into an overestimation of hypertension (white-coat hypertension) [9].

The *EURIKA study* [15] (European Study on CVD Risk Prevention and Management in Daily Practice [conducted in 12 European countries]) and *EUROASPIRE IV* [48] (European Action on Secondary and Primary Prevention by Intervention to Reduce Events [conducted in 14 European countries]) reported on inadequate medication control in 59% to 61% of hypertensives, 27% to 67% of patients with dyslipidemia, and in 41% (*EUROASPIRE*, HbA1c <7%) to 63% (*EURIKA*, HbA1c ≥6.5%) of diabetics across Europe. An observational study of 8928 patients from 320 general practices over 10 European countries assessed predictors for risk factor control in patients at high vascular risk. Predictors for poor risk factor control were female sex, living alone, and lower educational status [2].

### Risk factor control by absolute risk for fatal CVD

Applying SCORE, 35.6% of the participants were at medium and 7.5% at high to very high risk for CV death within the upcoming 10 years, probably mainly due to the age distribution of our population. Vascular risk factor control was worst amongst participants at highest risk of subsequent CVD. There are a few further patient cohort studies available which calculated the cardiovascular disease risk via SCORE. The German *EURIKA* study assessed the 10-year risk of CVD death in 7641 outpatients free from CVD aged ≥50 years (low vs. high CVD risk) and found a quite higher percentage of individuals at high risk (40.1%). With 22%, also higher proportions for participants at high risk was reported for the CoRiMa study compared to STAAB. In the *EURIKA* study about one-third of participants on pharmacotherapy was still at high CV risk [11, 15, 42], implying insufficient risk factor control. A Belgian survey conducted in primary care (*n* = 11,069) detected 38.0% individuals at high and 26.6% at very high risk [49].

The comparatively low number of individuals at high to very high risk in our population-based study (7.5%) indicates that the prevalence of subjects at high risk for CV death might be overestimated in studies including primary care patients because of different risk profiles such as a higher comorbidity burden in a sicker and older population. Further, CV risk factor detection might be much higher when there is a clinical suspicion of risk.

### Limitations

Owing to the study design and recruitment methodology, several limitations apply. We cannot exclude effects of selection bias, as only 32.6% of the invited population participated in the study. More healthy subjects might have had a higher motivation to participate in the STAAB program. Findings from our local Würzburg population may not be generalizable to other regions in Germany due to regional differences in age structure, risk factor profile, or lifestyle factor distribution. Due to the limited availability of original data from other studies, we were unable to provide direct age-standardized comparisons. We further did not assess, whether respective lifestyle advice had been given by the participants´ healthcare provider. Moreover we have no information about potential inflammation markers. Despite standardized measurements, single-occasion measurements (e.g., BP levels) may have led to amplified associations. Furthermore, the predictive model only accounted for measured factors leaving room for residual confounding.

## Conclusion

Our results derived from a German population-based sample indicate that there is considerable potential to improve adherence to CV prevention guidelines, particularly in the management of hypertension, DM, overweight, physical activity, and smoking. Considering the high values of unreported high BP and LDL cholesterol levels in younger age groups (30–39 years), a target for better guideline implementation might be a more comprehensive screening for vascular risk factors particularly in younger age groups of the general population. The association of age, male sex and educational level with a greater number of uncontrolled risk factors suggests that prevention strategies should also focus on individual patient needs considering specific explanations and close guidance from the physician’s side, in particular for specific subgroups such as people with lower educational level.

## Additional files

Additional file 1: **Table S1.** Definition of uncontrolled cardiovascular risk factors. Definition of the six uncontrolled cardiovascular risk factors (blood pressure, glycemic control, LDL cholesterol, tabaco abuse, physically inactive, overweight) and their subgroups (PDF 337kb)

Additional file 2: **Table S2.** Sensitivity analysis according to sex. Odds ratios (OR) (95%-CI) for 3–6 (referent: 0–2) insufficiently controlled cardiovascular risk factors adjusted for sociodemographic status stratified by sex (PDF 343 kb)

## Abbrevations

BMI: Body mass index
BP: Blood pressure
CARLA: Cardiovascular Disease, Living and Ageing in Halle Study in East Germany
CHFC: Comprehensive Heart Failure Center
CoRiMa: The German Coronary Risk Management
CVD: Cardiovascular disease
CVRF: Cardiovascular risk factor
DEGS1: German Health Interview and Examination Survey for Adults
DETECT: Diabetes Cardiovascular Risk-Evaluation: Targets and Essential Data for Commitment of Treatment
DIAB-CORE: The Diabetes Collaborative Research of Epidemiologic Studies
DM: Diabetes mellitus
EURIKA: European Study on CVD Risk Prevention and Management in Daily Practice
EUROASPIRE: European Action on Secondary and Primary Prevention by Intervention to Reduce Events
GNHIES98: National Health Interview and Examination Surveys 1998
HYDRA: Hypertension and Diabetes Risk Screening and Awareness Study
ICE-B: Institute of Clinical Epidemiology and Biometry
KORA: Cooperative Health Research in the Augsburg Region in South Germany
LDL cholesterol: Low density liproprotein
OR: Odds ratio
SCORE: Systematic Coronary Risk Evaluation
SD: Standard deviation
STAAB: Characteristics and Course of Heart Failure Stages A-B and Determinants of Progression

## References

[1] Bundesamt S (2013). Gestorbene nach ausgewählten Todesursachen.

[2] Ludt S, Wensing M, Campbell SM, Ose D, van Lieshout J, Rochon J, Uhlmann L, Szecsenyi J (2014). The challenge of cardiovascular prevention in primary care: implications of a European observational study in 8928 patients at different risk levels. Eur J Prev Cardiol.

[3] Lopez AD, Mathers CD, Ezzati M, Jamison DT, CJL M. Global and regional burden of disease and risk factors, 2001**:** Systematic analysis of population health data. Lancet. 367(9524):1747–57. 10.1016/S0140-6736(06)68770-9 16731270

[4] Piepoli MF, Hoes AW, Agewall S, Albus C, Brotons C, Catapano AL, Cooney MT, Corra U, Cosyns B, Deaton C, et al. European guidelines on cardiovascular disease prevention in clinical practice: the sixth joint task force of the European Society of Cardiology and Other Societies on cardiovascular disease prevention in clinical practice (constituted by representatives of 10 societies and by invited experts): developed with the special contribution of the European Association for Cardiovascular Prevention & rehabilitation (EACPR). Eur J Prev Cardiol. 2016:2016. 10.1177/2047487316653709 27353126

[5] Catapano AL, Graham I, De Backer G, Wiklund O, Chapman MJ, Drexel H, Hoes AW, Jennings CS, Landmesser U, Pedersen TR *et al*: 2016 ESC/EAS guidelines for the Management of Dyslipidaemias: the task force for the Management of Dyslipidaemias of the European Society of Cardiology (ESC) and European atherosclerosis society (EAS) developed with the special contribution of the European Assocciation for Cardiovascular Prevention & Rehabilitation (EACPR). Atherosclerosis 2016, 253:281–344. 10.1016/j.atherosclerosis.2016.08.018 27594540

[6] Liakos CI, Grassos CA, Babalis DK (2015). 2013 ESH/ESC guidelines for the management of arterial hypertension: what has changed in daily clinical practice. High blood pressure & cardiovascular prevention : the official journal of the Italian Society of Hypertension.

[7] Ryden L, Grant PJ, Anker SD, Berne C, Cosentino F, Danchin N, Deaton C, Escaned J, Hammes HP, Huikuri H (2014). ESC guidelines on diabetes, pre-diabetes, and cardiovascular diseases developed in collaboration with the EASD - summary. Diab Vasc Dis Res.

[8] Pieper L, Wittchen HU, Glaesmer H, Klotsche J, Marz W, Stalla G, Lehnert H, Zeiher AM, Silber S, Koch U (2005). Cardiovascular high-risk constellations in primary care. DETECT study 2003. Bundesgesundheitsblatt, Gesundheitsforschung, Gesundheitsschutz.

[9] Sharma AM, Wittchen HU, Kirch W, Pittrow D, Ritz E, Goke B, Lehnert H, Tschope D, Krause P, Hofler M (2004). High prevalence and poor control of hypertension in primary care: cross-sectional study. J Hypertens.

[10] Wittchen HU, Glaesmer H, Marz W, Stalla G, Lehnert H, Zeiher AM, Silber S, Koch U, Bohler S, Pittrow D (2005). Cardiovascular risk factors in primary care: methods and baseline prevalence rates--the DETECT program. Curr Med Res Opin.

[11] Geller JC, Cassens S, Brosz M, Keil U, Bernarding J, Kropf S, Bierwirth RA, Lippmann-Grob B, Schultheiss HP, Schluter K (2007). Achievement of guideline-defined treatment goals in primary care: the German coronary risk management (CoRiMa) study. Eur Heart J.

[12] Bramlage P, Pittrow D, Wittchen HU, Kirch W, Boehler S, Lehnert H, Hoefler M, Unger T, Sharma AM (2004). Hypertension in overweight and obese primary care patients is highly prevalent and poorly controlled. Am J Hypertens.

[13] Bramlage P, Wittchen HU, Pittrow D, Kirch W, Krause P, Lehnert H, Unger T, Hofler M, Kupper B, Dahm S (2004). Recognition and management of overweight and obesity in primary care in Germany. International journal of obesity and related metabolic disorders : journal of the International Association for the Study of Obesity.

[14] Lehnert H, Wittchen HU, Pittrow D, Bramlage P, Kirch W, Bohler S, Hofler M, Ritz E (2005). Prevalence and pharmacotherapy of diabetes mellitus in primary care. Deutsche medizinische Wochenschrift (1946).

[15] Banegas JR, Lopez-Garcia E, Dallongeville J, Guallar E, Halcox JP, Borghi C, Masso-Gonzalez EL, Jimenez FJ, Perk J, Steg PG (2011). Achievement of treatment goals for primary prevention of cardiovascular disease in clinical practice across Europe: the EURIKA study. Eur Heart J.

[16] Tokgozoglu L, Bruckert E (2012). Implementation, target population, compliance and barriers to risk guided therapy. Eur J Prev Cardiol.

[17] Wagner M, Tiffe T, Morbach C, Gelbrich G, Stork S, Heuschmann PU. Characteristics and course of heart failure stages A-B and determinants of progression - design and rationale of the STAAB cohort study. Eur J Prev Cardiol. 2016; 10.1177/2047487316680693 27879413

[18] Craig CL, Marshall AL, Sjostrom M, Bauman AE, Booth ML, Ainsworth BE, Pratt M, Ekelund U, Yngve A, Sallis JF (2003). International physical activity questionnaire: 12-country reliability and validity. Med Sci Sports Exerc.

[19] Tabenkin H, Eaton CB, Roberts MB, Parker DR, McMurray JH, Borkan J (2010). Differences in cardiovascular disease risk factor management in primary care by sex of physician and patient. Ann Fam Med.

[20] Cordero A, Alegria E (2006). Sex differences and cardiovascular risk. Heart.

[21] Kaplan GA, Keil JE (1993). Socioeconomic factors and cardiovascular disease: a review of the literature. Circulation.

[22] Psaltopoulou T, Hatzis G, Papageorgiou N, Androulakis E, Briasoulis A, Tousoulis D (2017). Socioeconomic status and risk factors for cardiovascular disease: impact of dietary mediators. Hellenic journal of cardiology : HJC = Hellenike kardiologike epitheorese.

[23] Rucker V, Keil U, Fitzgerald AP, Malzahn U, Prugger C, Ertl G, Heuschmann PU, Neuhauser H (2016). Predicting 10-year risk of fatal cardiovascular disease in Germany: an update based on the SCORE-Deutschland risk charts. PLoS One.

[24] Conroy RM, Pyorala K, Fitzgerald AP, Sans S, Menotti A, De Backer G, De Bacquer D, Ducimetiere P, Jousilahti P, Keil U (2003). Estimation of ten-year risk of fatal cardiovascular disease in Europe: the SCORE project. Eur Heart J.

[25] Gosswald A, Lange M, Dolle R, Holling H (2013). The first wave of the German health interview and examination survey for adults (DEGS1): participant recruitment, fieldwork, and quality management. Bundesgesundheitsblatt, Gesundheitsforschung, Gesundheitsschutz.

[26] Lowel H, Doring A, Schneider A, Heier M, Thorand B, Meisinger C (2005). The MONICA Augsburg surveys--basis for prospective cohort studies. Gesundheitswesen (Bundesverband der Arzte des Offentlichen Gesundheitsdienstes (Germany)).

[27] Volzke H (2012). Study of health in Pomerania (SHIP). Concept, design and selected results. Bundesgesundheitsblatt, Gesundheitsforschung, Gesundheitsschutz.

[28] Greiser KH, Kluttig A, Schumann B, Kors JA, Swenne CA, Kuss O, Werdan K, Haerting J (2005). Cardiovascular disease, risk factors and heart rate variability in the elderly general population: design and objectives of the CARdiovascular disease, living and ageing in Halle (CARLA) study. BMC Cardiovasc Disord.

[29] Holle R, Happich M, Lowel H, Wichmann HE (2005). KORA--a research platform for population based health research. Gesundheitswesen (Bundesverband der Arzte des Offentlichen Gesundheitsdienstes (Germany)).

[30] Diederichs C, Neuhauser H (2017). The incidence of hypertension and its risk factors in the German adult population: results from the German National Health Interview and examination survey 1998 and the German health interview and examination survey for adults 2008-2011. J Hypertens.

[31] Ruckert IM, Schunk M, Holle R, Schipf S, Volzke H, Kluttig A, Greiser KH, Berger K, Muller G, Ellert U (2012). Blood pressure and lipid management fall far short in persons with type 2 diabetes: results from the DIAB-CORE consortium including six German population-based studies. Cardiovasc Diabetol.

[32] Bohley S, Kluttig A, Werdan K, Nuding S, Greiser KH, Kuss O, Markus MR, Schmidt CO, Volzke H, Krabbe C (2016). Changes of individual perception in psychosocial stressors related to German reunification in 1989/1990 and cardiovascular risk factors and cardiovascular diseases in a population-based study in East Germany. BMJ Open.

[33] Schipf S, Werner A, Tamayo T, Holle R, Schunk M, Maier W, Meisinger C, Thorand B, Berger K, Mueller G (2012). Regional differences in the prevalence of known type 2 diabetes mellitus in 45-74 years old individuals: results from six population-based studies in Germany (DIAB-CORE consortium). Diabetic medicine : a journal of the British Diabetic Association.

[34] Laxy M, Knoll G, Schunk M, Meisinger C, Huth C, Holle R (2016). Quality of diabetes Care in Germany Improved from 2000 to 2007 to 2014, but improvements diminished since 2007. Evidence from the population-based KORA studies. PLoS One.

[35] Ruckert IM, Baumert J, Schunk M, Holle R, Schipf S, Volzke H, Kluttig A, Greiser KH, Tamayo T, Rathmann W (2015). Blood pressure control has improved in people with and without type 2 diabetes but remains suboptimal: a longitudinal study based on the German DIAB-CORE consortium. PLoS One.

[36] Scheidt-Nave C, Du Y, Knopf H, Schienkiewitz A, Ziese T, Nowossadeck E, Gosswald A, Busch MA (2013). prevalence of dyslipidemia among adults in Germany: results of the German health interview and examination survey for adults (DEGS 1). Bundesgesundheitsblatt, Gesundheitsforschung, Gesundheitsschutz.

[37] Mensink GB, Schienkiewitz A, Haftenberger M, Lampert T, Ziese T, Scheidt-Nave C (2013). Overweight and obesity in Germany: results of the German health interview and examination survey for adults (DEGS1). Bundesgesundheitsblatt, Gesundheitsforschung, Gesundheitsschutz.

[38] Krug S, Jordan S, Mensink GB, Muters S, Finger J, Lampert T (2013). Physical activity: results of the German health interview and examination survey for adults (DEGS1). Bundesgesundheitsblatt, Gesundheitsforschung, Gesundheitsschutz.

[39] Lampert T, von der Lippe E, Muters S (2013). Prevalence of smoking in the adult population of Germany: results of the German health interview and examination survey for adults (DEGS1). Bundesgesundheitsblatt, Gesundheitsforschung, Gesundheitsschutz.

[40] Völzke H, Ittermann T, Schmidt CO, Baumeister SE, Schipf S, Alte D, Biffar R, John U, Hoffmann W (2015). Prevalence trends in lifestyle-related risk factors. Dtsch Arztebl International.

[41] Landgraf J, Wishner SH, Kloner RA (2010). Comparison of automated oscillometric versus auscultatory blood pressure measurement. Am J Cardiol.

[42] Schmieder RE, Goebel M, Bramlage P (2012). Barriers to cardiovascular risk prevention and management in Germany--an analysis of the EURIKA study. Vasc Health Risk Manag.

[43] Huntink E, Wensing M, Klomp MA, van Lieshout J (2015). Perceived determinants of cardiovascular risk management in primary care: disconnections between patient behaviours, practice organisation and healthcare system. BMC Fam Pract.

[44] Balder JW, Scholtens S, de Vries JK, van Schie LM, Boekholdt SM, Hovingh GK, Kamphuisen PW, Kuivenhoven JA (2015). Adherence to guidelines to prevent cardiovascular diseases: the LifeLines cohort study. Neth J Med.

[45] Wiersma T, Smulders YM, Stehouwer CD, Konings KT, Lanphen J (2012). summary of the multidisciplinary guideline on cardiovascular risk management (revision 2011). Ned Tijdschr Geneeskd.

[46] Koopman C, Vaartjes I, Heintjes EM, Spiering W, van Dis I, Herings RM, Bots ML (2013). Persisting gender differences and attenuating age differences in cardiovascular drug use for prevention and treatment of coronary heart disease, 1998-2010. Eur Heart J.

[47] Barbero U, Scacciatella P, Iannaccone M (2017). Culprit plaque characteristics in younger versus older patients with acute coronary syndromes: an optical coherence tomography study from the FORMIDABLE registry.

[48] Kotseva K, De Bacquer D, De Backer G, Ryden L, Jennings C, Gyberg V, Abreu A, Aguiar C, Conde AC, Davletov K, et al. Lifestyle and risk factor management in people at high risk of cardiovascular disease. A report from the European Society of Cardiology European Action on secondary and primary prevention by intervention to reduce events (EUROASPIRE) IV cross-sectional survey in 14 European regions. Eur J Prev Cardiol. 2016; 10.1177/2047487316667784 27638542

[49] Missault L, Witters N, Imschoot J (2010). High cardiovascular risk and poor adherence to guidelines in 11,069 patients of middle age and older in primary care centres. European journal of cardiovascular prevention and rehabilitation : official journal of the European Society of Cardiology, Working Groups on Epidemiology & Prevention and Cardiac Rehabilitation and Exercise Physiology.

