# Supplementary material for: Control of cardiovascular risk factors and its determinants in the general population– findings from the STAAB cohort study
Source: BMC Cardiovasc Disord. 2017 Nov 2;17:276. doi: 10.1186/s12872-017-0708-x (PMC5669014; doi:10.1186/s12872-017-0708-x)
Supplement: Supplementary file 1 — Table S1. Definition of uncontrolled cardiovascular risk factors. Definition of the six uncontrolled cardiovascular risk factors (blood pressure, glycemic control, LDL cholesterol, tabaco abuse, physically inactive, overweight) and their subgroups (PDF 337kb) [file 12872_2017_708_MOESM1_ESM.pdf]

Table 4: Definition of uncontrolled risk factors

Uncontrolled risk factor	Definition
Blood Pressure	
High BP level despite medication	≥140/90 mmHg & antihypertensive medication
High BP level despite medication in diabetics	
Type 1	≥140/85 mmHg & antihypertensive medication & self-reported type 1 diabetes & antidiabetic medication
Type 2	≥130/80 mmHg & antihypertensive medication & self-reported type 2 diabetes & antidiabetic medication
Glycemic control	
HbA1c ≥ 7% despite medication	HbA1c ≥7% & antidiabetic medication
LDL cholesterol	
High LDL-C level despite medication	≥115 mg/dl & lipid-lowering agent
High LDL-C level despite medication in diabetics	≥100 mg/dl & lipid-lowering agent & antidiabetic medication
Tabacco use	Self-reported in an structured interview
Physically inactive	<150 min/week moderate activity or < 75 min/week strenuous activity
Overweight	
Despite physically activity	Body mass index >25 kg/m² & physically active
