# Supplementary material for: Control of cardiovascular risk factors and its determinants in the general population– findings from the STAAB cohort study
Source: BMC Cardiovasc Disord. 2017 Nov 2;17:276. doi: 10.1186/s12872-017-0708-x (PMC5669014; doi:10.1186/s12872-017-0708-x)
Supplement: Supplementary file 2 — Table S2. Sensitivity analysis according to sex. Odds ratios (OR) (95%-CI) for 3–6 (referent: 0–2) insufficiently controlled cardiovascular risk factors adjusted for sociodemographic status stratified by sex (PDF 343 kb) [file 12872_2017_708_MOESM2_ESM.pdf]

Table 5: Sensitivity analysis according to sex

Variables	Female		Male	
	**OR (95%-KI)**	**p-value**	**OR (95%-KI)**	**p-value**
**Age groups in years**		< 0.001		0.04
30-39	1		1	
40-49	1.48 (0.52; 4.12)		2.09 (1.06; 4.12)	
50-59	3.81 (1.37; 10.58)		1.43 (0.70; 2.93)	
60-69	4.73 (1.66; 13.47)		2.52 (1.25; 5.10)	
70-79	8.04 (2.27; 28.53)		2.65 (1.08; 6.63)	
**Highest education in year**		<0.01		0.06
Tertiary	1		1	
Secondary	1.20 (0.70; 2.08)		1.45 (0.90; 2.35)	
Primary	2.68 (1.45; 4.96)		1.71 (1.07; 2.75)	
**Marital Status***		0.604		0.21
Married	1		1	
Single	0.64 (0.32; 1.28)		0.88 (0.53; 1.46)	
Divorced	0.85 (0.40; 1.78)		1.73 (0.85; 3.51)	
Widowed	1.05 (0.44; 2.49)		0.41 (0.10; 1.63)	

*OR given just before removing from model
